# Safety and efficacy of cytokine-induced killer cells for gastric cancer: a systematic review and meta-analysis

**DOI:** 10.3389/fonc.2026.1834073

**Published:** 2026-06-08

**Authors:** Mingming Zhu, Changlong Yang, Bingqian Liang, Qin Liu

**Affiliations:** Department of Gastrointestinal Surgery, Yunnan Cancer Hospital, The Third Affiliated Hospital of Kunming Medical University, Peking University Cancer Hospital, Kunming, Yunnan, China

**Keywords:** cytokine-induced killer cell, gastric cancer, immunotherapy, meta-analysis, systematic review

## Abstract

**Background:**

Cytokine-induced killer cell (CIK) cell therapy shows promising antitumor effects in gastric cancer (GC). Given variability in clinical outcomes across studies, a systematic review and meta-analysis was conducted to assess overall safety and efficacy.

**Methods:**

Three databases (PubMed, Scopus, and Web of Science) were searched for studies evaluating the safety and efficacy of CIK/dendritic cell-cytokine-induced killer cell (DC-CIK) cell therapy in gastric cancer. Time-to-event outcomes (OS, DFS, PFS) were synthesized using hazard ratios (HRs); fixed time-point survival effects from 6 months to 5 years were additionally summarized in supplementary analyses. Fixed- or random-effects models were applied as appropriate. Publication bias was examined using Begg’s test and funnel plots, and robustness was explored with the Trim-and-Fill method. Safety and secondary outcomes (e.g., adverse events and T-lymphocyte subsets) were summarized descriptively.

**Results:**

A total of 22 trials including 2,149 patients were included. After risk-of-bias assessment, 13 studies were included in the quantitative synthesis. Pooled HRs showed significant benefits of CIK/DC-CIK plus chemotherapy compared with chemotherapy alone: OS (9 studies; 1,047 patients) HR 0.60 (95% CI 0.48–0.75; p<0.001), PFS (4 studies; 418 patients) HR 0.60 (95% CI 0.46–0.77; p<0.001), and DFS (4 studies; 523 patients) HR 0.70 (95% CI 0.58–0.86; p<0.001). ORR showed a non-significant trend in favor of CIK/DC-CIK (3 studies; 225 patients; pooled log OR 0.46, 95% CI −0.07 to 0.99; p=0.09), while DCR was significantly improved (3 studies; 225 patients; pooled log OR 0.81, 95% CI 0.17 to 1.45; p=0.01). Immunological analyses showed increased CD3+, CD4+, and CD4+/CD8+ T cells, with a slight decrease in CD8+ cells. Fever was the most frequently reported infusion-related adverse event, and no fatal adverse events were reported; however, grade ≥3 adverse events were inconsistently reported and could not be pooled, limiting certainty regarding comparative safety. No geographic restriction was applied; however, all identified eligible studies were conducted in China.

**Conclusion:**

Based on available studies, CIK/CIK-DC immunotherapy combined with chemotherapy significantly improves OS, PFS, and DFS in patients with gastric cancer and increases the DCR, without inducing severe adverse effects. Although these findings suggest potential benefit, validation in more diverse populations is needed.

**Systematic review registration:**

https://www.crd.york.ac.uk/PROSPERO/, identifier CRD420251160258.

## 1 Introduction

Gastric cancer (GC) remains one of the leading causes of cancer-related mortality worldwide, ranking as the fifth most commonly diagnosed malignancy and the fourth leading cause of cancer death as of 2020, with over one million new cases and approximately 769,000 deaths annually (1). The disease burden is particularly high in East Asia, Eastern Europe, and parts of Latin America, where Helicobacter pylori infection- a primary risk factor- is more prevalent. Despite advances in diagnosis and therapy, the prognosis for gastric cancer remains poor, especially when diagnosed at an advanced stage. The 5-year overall survival rate varies significantly by region and stage at diagnosis, ranging from over 60% in countries with effective screening programs like Japan, to less than 30% in many Western nations where diagnosis often occurs at a later stage (2). This disparity underscores the urgent need for improved early detection strategies, equitable healthcare access, and targeted treatments to enhance patient outcomes globally.

Over the past decade, immunotherapy has transformed the landscape of cancer treatment, offering promising clinical benefits across a wide range of solid and hematological malignancies (3). Among these, immune checkpoint inhibitors have shown significant efficacy, particularly in tumors characterized by a high tumor mutational burden or microsatellite instability (4). In the context of GC, immune checkpoint inhibitors such as nivolumab and pembrolizumab have demonstrated improved survival outcomes, particularly in high microsatellite instability or PD-L1-positive cases and are now incorporated into standard treatment regimens for advanced disease (5–7).

Beyond the above treatments, adoptive cell immunotherapy is gaining attention for its potential to treat solid tumors, including gastric cancer. One of the most actively studied approaches is Cytokine-Induced Killer (CIK) cell therapy, an adoptive immunotherapy derived from autologous peripheral blood mononuclear cells (PBMCs), expanded ex vivo under cytokine stimulation. This heterogeneous population includes T cells (CD3^+^CD56^-^), NK cells (CD3^-^CD56^+^), and NK-like T cells (CD3^+^CD56^+^), the latter believed to be the principal effectors of tumor cell cytotoxicity in a non-MHC-restricted manner (8, 9).

Although no adoptive immunotherapy, including CIK therapy, has received FDA approval for gastric cancer, it has been widely investigated in East Asia, particularly in China, where several clinical trials have reported enhanced survival outcomes and reduced recurrence rates when CIK therapy is used as an adjuvant or palliative treatment (10–12). For instance, combining CIK therapy with conventional chemotherapy or dendritic cell (DC) therapy, known as DC-CIK therapy, has been shown to potentiate anti-tumor immunity and improve disease control in patients with advanced GC (12).

The safety profile of CIK therapy is generally favorable, with low incidence of severe adverse events, making it a viable adjunct to chemotherapy in both postoperative and palliative settings. Numerous clinical studies, predominantly conducted in China, including randomized controlled trials, have evaluated the efficacy of CIK therapy in gastric cancer (10, 13). However, the heterogeneity in study designs and endpoints has limited the integration of findings into clinical practice guidelines.

Therefore, a comprehensive systematic review and meta-analysis is warranted to assess the clinical efficacy of CIK therapy, alone or in combination with chemotherapy or DC therapy, in the treatment of GC, consolidating both English and Chinese literature to inform future research and clinical translation.

## 2 Methods

In this systematic review, we followed the Preferred Reporting Items for Systematic Reviews and Meta-Analyses (PRISMA) guidelines (14). The protocol of this systematic review and meta-analysis was submitted to PROSPERO (CRD420251160258). The research question was defined according to the PICOS framework as follows: Population: patients with gastric cancer of any reported clinical stage; Intervention: chemotherapy combined with CIK or DC-CIK immunotherapy; Comparator: chemotherapy alone; Outcomes: overall survival, progression-free survival, disease-free survival, objective response rate, disease control rate, adverse events, and immune-cell subset changes; Study design: randomized and non-randomized clinical trials.

### 2.1 Literature search and study selection

A comprehensive literature search with a pre-defined search strategy was conducted from database inception up to September 2025 across three databases, including PubMed, Web of Science, and Scopus. This search was further supplemented by manual searching in Google Scholar and by screening the reference lists of relevant review articles to identify additional primary studies. The search terms included “Gastric Carcinoma” OR “Gastric Cancer” OR “Gastric Tumor” OR “Gastric Neoplasm” OR “Stomach Carcinoma” OR “Stomach Cancer” OR “Stomach Tumor” OR “Stomach Neoplasm” combined with “Cytokine Induced Killer Cell” OR “Cytokine-Induced Killer Cell” OR “CIK” OR “Cell Lymphocyte-Activated Killer Cell” OR Lymphocyte Activated Killer Cell”. No restrictions were applied regarding language, publication year, or publication status. Studies published in Chinese were eligible; data extraction and translation of relevant information were performed by native Chinese-speaking authors. The full database-specific search strategies are provided in **Supplementary Table 1**.

After removing duplicates, two independent reviewers (MZ, and CY) screened the titles and abstracts of the identified records. Full-text articles were then assessed for inclusion, with any disagreements resolved through discussion or by consulting a third reviewer (QL).

### 2.2 Inclusion criteria

We included clinical trials of patients with GC of any reported clinical stage (including localized, locally advanced, recurrent, or metastatic disease, as defined in the original studies), in which intervention group received chemotherapy combined with CIK or DC-CIK immunotherapy and the control group received chemotherapy alone. Studies must have reported at least one efficacy or safety outcome to be included in this systematic review. Studies were excluded if they were non-primary articles (e.g., reviews, letters, case reports), lacked the required intervention/comparator design, did not enroll patients with gastric cancer, or did not report relevant efficacy or safety outcomes. Review articles were not eligible for inclusion as studies in the systematic review; however, their reference lists were screened during manual searching to identify potentially eligible primary clinical studies. In case of multiple studies published on the same population, the most recent and complete study was included.

### 2.3 Data extraction

Initially, relevant data were extracted by two authors (MZ, and CY) and subsequently verified by a third author (QL). For studies published in Chinese, native Chinese-speaking authors conducted the data extraction and translated the content into English for inclusion in the analysis. This data collection followed a prepared checklist that included first author, publication year, study design, sample size, number of patients in the treatment and control groups, gender, age, follow-up period, Eastern Cooperative Oncology Group (ECOG) performance/Karnofsky Performance Scale (KPS), stage of cancer, metastasis, CIK dosage, cycles of CIK therapy, perfusion/infusion-interval, cycle duration, concomitant chemotherapy/adjuvant therapy, overall survival rate (OS), progression-free survival rate (PFS), disease-free survival rate (DFS) (including 6-month, 1-year, 18-month, 2-year, 3-year, 4-year and 5 years survival rates), objective response rate (ORR), disease control rate (DCR), adverse events, and T lymphocyte subsets changes (e.g. CD3, CD4, CD8, CD4/CD8, and NK).

### 2.4 Statistical analysis

The meta-analysis was conducted using Stata 17.0 (StataCorp, TX, USA). For time-to-event outcomes (OS, PFS, and DFS), hazard ratios (HRs) with corresponding 95% confidence intervals (CIs) were used as the primary effect size. When HRs were not directly reported, we derived log(HR) and its standard error using established approaches for incorporating published survival data, including extraction from Kaplan–Meier curves and reconstruction from available summary statistics (15, 16). Pooled HRs were calculated using inverse-variance weighting, applying a random-effects model when between-study heterogeneity was present.

Because a single HR implicitly assumes proportional hazards and crossing Kaplan–Meier curves or clearly time-varying hazards may render an overall HR difficult to interpret, we additionally summarized fixed time-point survival (e.g., 6-month to 5-year OS/PFS/DFS) as secondary analyses, reporting effect estimates at prespecified time points in the **Supplementary File**.

The potential heterogeneity across studies was examined via Cochran’s Q-statistic and I^2^ statistics to determine the most suitable model (heterogeneity was considered at P < 0.1 or I^2^ > 60%). The fixed or random-effects models were used to perform the analysis accordingly. When heterogeneity existed, a random-effects model with the restricted maximum-likelihood (REML) method was used; otherwise, a fixed-effects model with the inverse-variance method was used. Following the analysis, heterogeneity across studies was further evaluated using the I² statistic. An I² value of ≤30% was considered indicative of low heterogeneity, 30–60% as moderate, and ≥60% as high heterogeneity.

Subgroup analyses were conducted for the primary outcome (OS). The analyses included (1): comparison of CIK therapy alone versus DC-CIK combination therapy, and (2) comparison based on study design, i.e., RCT versus non-RCT.

In meta‐regression exploring potential sources of heterogeneity in the primary survival analysis, immune‐phenotype changes emerged as significant moderators of the treatment effect. Specifically, greater post-treatment increases in NK cells, CD4+ T cells, and CD3+ T cells were each associated with a more favorable survival effect. NK change showed a significant inverse association with the effect size (β = −0.043, SE = 0.02; p = 0.032; 95% CI −0.0830 to −0.0037; 4 studies). Similarly, CD4 change (β = −0.0627, SE = 0.0253; p = 0.013; 95% CI −0.1124 to −0.0131; 5 studies) and CD3 change (β = −0.0226, SE = 0.00915; p = 0.014; 95% CI −0.0405 to −0.00465; 5 studies) were significant, and each model explained a large proportion of between-study variability with minimal residual heterogeneity. These findings suggest that studies showing stronger immune activation (higher CD3/CD4/NK increases) tended to report larger survival benefits with CIK/DC-CIK therapy. However, because these regressions were based on a small number of studies (n = 4–5), the results should be interpreted cautiously. Other prespecified moderators (CIK dosage, gender ratio, mean age, baseline metastasis percentage, CD8 change, and CD4/CD8 change) were not consistently significant, and subgroup comparisons by CIK vs. DC-CIK and RCT vs. non-RCT did not demonstrate clear effect modification.

To assess potential publication bias, a funnel plot was generated for the outcome with the largest number of reported studies (OS). Begg’s and Egger’s tests were also applied to statistically evaluate publication bias. Additionally, the Trim-and-Fill method was employed to estimate the impact of potential publication bias on the pooled effect size. A p-value of less than 0.05 was considered statistically significant. For sensitivity analysis, the robustness of the overall effect was examined by sequentially removing one study at a time.

### 2.5 Quality assessment

Study quality was assessed according to the Cochrane Handbook, evaluating seven domains of potential bias (1): random sequence generation (selection bias) (2), allocation concealment (selection bias) (3), blinding of participants and personnel (performance bias) (4), blinding of outcome assessment (detection bias) (5), incomplete outcome data (attrition bias) (6), selective reporting (reporting bias), and (7) other sources of bias. Each domain was rated as “low risk,” “unclear risk,” or “high risk” of bias (17). Two authors (MZ, and CY) independently completed the assessments. Any disagreements were resolved with the input of a third author (QL).

Study screening, extraction, and risk-of-bias assessment were performed independently by two reviewers, with disagreements resolved by consensus/third reviewer, per the PROSPERO-registered protocol.

### 2.6 Certainty of evidence using GRADE assessment

The certainty of evidence for the primary outcome (overall survival) was evaluated using the Grading of Recommendations Assessment, Development and Evaluation (GRADE) approach. The body of evidence was assessed across the five GRADE domains: risk of bias, inconsistency, indirectness, imprecision, and publication bias, and an overall certainty rating (high, moderate, low, or very low) was assigned for OS. Evidence from randomized trials was initially rated as high certainty and downgraded when serious concerns were identified in any domain. Two reviewers independently performed the GRADE assessment and resolved disagreements through discussion or with input from a third reviewer when needed.

## 3 Results

Through electronic database searching, we identified 1,019 records (PubMed n = 254, Web of Science n = 244, Scopus n = 521). After removing duplicates (n = 209), 810 records were screened by title/abstract, and 149 full-text reports were assessed for eligibility, yielding 17 included studies from database searching. In addition, 8 records were identified via other methods (Google Scholar n = 2; citation searching n = 6), of which 5 studies were included after full-text assessment. Overall, 22 studies were included (10–13, 18–35) were included in this systematic review.

Specifically, we systematically assessed overlap across studies using predefined indicators, including the names of authors and their affiliations, recruiting center/hospital, recruitment period, inclusion criteria, intervention regimen, baseline demographics, and sample size patterns. If multiple reports were judged to arise from the same underlying cohort, we treated them as one study unit and prioritized the report with the largest sample size and the most recent report. We identified potential overlap between Jiang 2006, Jiang 2008, Jiang 2010, and Shi 2012 studies and included the Shi 2012 study for OS, DFS, and PFS outcomes as the most recent study with the largest sample size. Then, for ORR, and DCR outcomes, since Shi et al. did not report any results on ORR and DCR, we included Jiang 2008. In addition, since Cui published two papers in 2014 and 2015, we included the Cui 2015 paper for meta-analysis.

**Figure 1:**
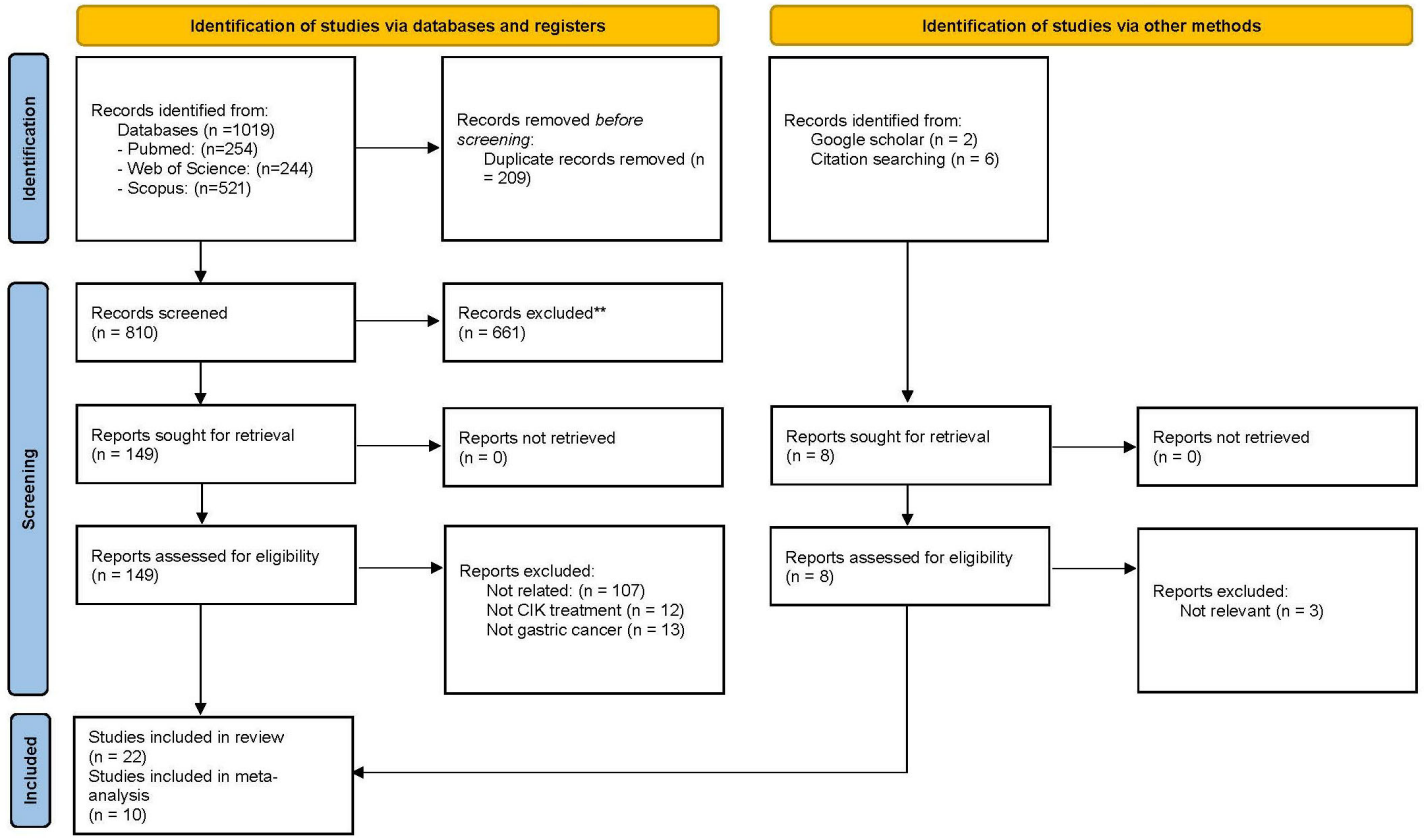
PRISMA 2020 flow diagram showing the identification and screening of databases, eligibility assessment, and inclusion of studies.

After removing the above-mentioned studies (three studies), careful quality assessment, and excluding the high-risk of bias studies (nine studies), 10 studies were finally included in the meta-analysis. The study selection process is shown in the PRISMA 2020 flow diagram (**Figure 1**).

### 3.1 Study and patient characteristics

The main characteristics of studies including first author, year, study design, follow-up, sample size, male/female ratio and age in each group, ECOG/KPS, cancer stage, metastasis, CIK-related outcomes (i.e. dosage, cycles, perfusion/cycle, interval, and cycle duration), concomitant drugs and adjuvant therapy, and median OS, PFS and DFS in each group are summarized in **Table 1**. All studies were performed in China. 14 out of the total 22 studies were RCT studies. Overall, 2149 patients with GC, 972 in the CIK/DC-CIK therapy (treatment) group, and 1177 in the non-CIK Therapy (control) group were available for analysis. For studies that provided the patient’s gender, 1182 out of 1666 patients (70.94%) were males (male/total: 524/756 in the treatment group, and 658/910 in the control group). Patients with GC diagnosed with all cancer stages were considered for analysis. In 7 studies, patients in the intervention arm received DC-CIK therapy, while in the remaining, CIK therapy was solely administered. Chemotherapy was the most common co-treatment with CIK/DC-CIK therapy, being used in all included studies. Chemotherapy regimens have been shown in detail in **Table 1**. Toxicity during the study intervention was reported by 14 studies, with the majority of the data being provided descriptively. Many of the described side effects were thought to be related to chemotherapy administered together with CIK/DC-CIK therapy, including bone marrow suppression, nausea, vomiting, neuropathy, diarrhea, and liver dysfunction. Fever was the most frequently reported adverse event associated with CIK/DC-CIK infusion (**Table 1**).

**Table 1:** Characteristics of included studies.

First Author, year	Study design; Follow‐up (months)	Sample size	Treatment (M, F)/control (M, F)	Male/female (treatment group); male/female (control group)	Age (treatment/control group)	ECOG; KPS	Stage, metastasis	Treatment	CIK dosage (×10–^9^ cells), cycles, perfusion/cycle, interval; cycle duration	Concomitant drugs; Adjuvant therapy	Adverse events (%)	Median OS (treatment/group); Median PFS (treatment/group); Median DFS (treatment/group)
Chen, 2015 (18)	CT; median: 44.1	226	89/137	70/19; 108/29	NA: NA	NA; NA	II, III	CIK	10; 3; NA; NA; 4 weeks	5-FU or capecitabin (4 cycle); gastrectomy	Treatment group: fever/fatigue: 14.6, rash: 5.6, diarrhea: 4.3	45/44; NA/NA; 41/32
Cui, 2014 (19)	CT; NM	46	25/21	NA; NA	NA; NA	NM; >70	NM	CIK	NA; NA; NA; NA; NA	Xelox; NA	NA	7.1/5.9; 4.8/3.1; NA/NA
Cui, 2015 (20)	RCT; median: 22.5, range: 10-51	58	30/28	20/10; 21/7	58.5 (median); 58 (median)	≤2; NM	I-IV	CIK	2.4-4; 6; 6; NA; 1 week	STG or CTG; radical surgery, palliative gastrectomy	Treatment group: Fever: 16.66	NR/NR; NR/16; NA/NA
Fan, 2012 (21)	Retrospective; NM	195	65/130	NA; NA	NA; NA	NA; NA	I-IV	CIK	10; 4-8; NA; NA; NA	Platinum + FU; NA	NA	96/32; 36/23; NA/NA
Gao, 2014 (22)	RCT; 98	54	27/27	16/11; 16/11	61.5 ± 12.8; 64.4 ± 12.77	NA; NA	I, II, III	CIK	5.88; 1-2; 3-5; NA; 2 weeks	low dose chemotherapy; surgery	Treatment group: Fever: 33	NR/30: NA/NA; NR/30
Jiang, 2006 (23)	RCT; 54	57	32/25	21/11; 18/7	54 (median); 52 (median);	NA; NA	IV	CIK	1; NA; 5; every other day; NA	Folfox4; palliative gastrectomy	Treatment group: Chill: 9.375, fever: 43.75, nausea: 6.25	6/6; NA/NA; NA/NA
Jiang, 2008 (24)	CT; NM	60	29/31	21/8; 24/7	66 (median); 67 (median)	NA; NA	II-IV	CIK	1; NA; NA; NA; 1; NA	FOLFOX4; NA	Treatment group: Chills: 44.82, fever: 31.03, dizziness & headache: 10.34, nausea & vomiting: 3.44, malaise: 10.34, injection site infection: 3.44, upper respiratory tract infection symptoms: 10.34, eye symptoms: 6.89	NA/NA; NA/NA; NA/NA
Jiang, 2010 (25)	RCT; 112	156	75/81	60/15; 62/19	62.4 ± 10.8; 59.9 ± 10.5	NA; NA	I - IV	CIK	1; 1-25; NA; NA; NA	Oxaliplatin + 5-FU; surgery	NA	49/27; NA/NA; NA/NA
Li, 2017 (26)	Retrospective; 24	92	46/46	35/11; 37/9	59 ± 9.2; 60 ± 7.3	NA; NA	I, II, III	CIK	5-10; 5; 1-15; NA; NA; 2–4 weeks	5-FU; radical surgery	NA	NR/NR; NA/NA; NR/35
Liu, 2013 (27)	RCT; 36	98	51/47	34/17; 31/16	56.1 ± 11.9; 55.2 ± 12.7	NA; NA	I - IV	CIK	1; NA; NA; every second day; NA	FOLFOX4 (2 cycle) + 5HT receptor blocker+ vitamin B6; surgery	NA	NR/NR; NR/NR; NA/NA
Liu, 2022 (10)	RCT; 24	106	53/53	33/20; 27/26	61.5 ± 9.0/63.1 ± 9.19	≤2; NA	III	DC-CIK	NA; 2; 2; NA; 4 weeks	oxaliplatin-5-fluorouracil; No	Treatment group: Fever: 56.6, hyperpyrexia and shivering: 17, rash: 7.5, myelosuppression: 5.7 Control group: Fever: 60.4, hyperpyrexia and shivering: 18.9, rash: 5.7, myelosuppression: 17	23.4/18.6; NA/NA; NA/NA
Lv, 2015 (28)	RCT; 22	72	37/35	22/15; 21/14	53 (median); 54 (median)	NA; NA	IV	DC-CIK	1; 2; 5; once every other day; NA	Folfox4; NA	Treatment group: Chills: 2.70, fever: 10.81, headache: 2.70, nausea and vomiting: 5.40	NA/NA; NA/NA; NA/NA
Ma, 2023 (11)	RCT; 44.25	59	31/28	25/6; 21/7	58 ± 3; 60 ± 4	<2, NA	III, IV	DC-CIK	8.6; 5; 1; NA; 3 weeks	S-1+oxaliplatin; NA	Treatment group: neutropenia: 12.9, anemia: 3.2, thrombocytopenia: 9.7, elevated total bilirubin: 3.2, elevated direct bilirubin: 3.2, leucopenia: 48.4, febrile neutropenia: 9.7, fever: 9.7, nausea: 61.3, vomiting: 29, decreased appetite: 64.5, diarrhea: 0, elevated ALT: 32.3, elevated AST: 29, peripheral neurotoxicity: 6.5, elevated Cr: 9.7, fatigue: 3.2 Control group: neutropenia: 35.7, anemia: 7.1, thrombocytopenia: 3.6, elevated total bilirubin: 3.6, elevated direct bilirubin: 3.6, leucopenia 75, febrile neutropenia: 32.1, fever: 7.1, nausea: 78.6, vomiting: 42.9, decreased appetite: 78.6, diarrhea: 7.1, elevated ALT: 35.7, elevated AST: 50, peripheral neurotoxicity: 17.9, elevated Cr: 0, fatigue: 3.6	17.8/9.75; 6.9/4.9; NA/NA
Mu, 2016 (29)	RCT; 24	28	13/15	10/3; 10/5	NA; NA	NA; NA	III, IV	DC-CIK	3; 3; 3; day 1-3;4 weeks	FOLFOX4/docetaxel; NA	Treatment group: nausea and vomiting: 30.8, thrombocytopenia: 23.1, neutropenia: 15.4, diarrhea: 15.4: Fever: 38.5 Control group: nausea and vomiting: 66.7, thrombocytopenia: 33.3, neutropenia: 26.7, diarrhea: 20, fever: 6.7	24/16; 22/16; NA/NA
Qiao, 2019 (12)	CT; 13.7	35	17/18	7/10; 6/12	61.8 ± 13.7; 60.1 ± 11.4	<2; NM	III, IV	DC-CIK	2.8; 2; 3; day 15, 17, 19; 3 weeks	S-1/+ cisplatin; NA	Treatment group: leucopenia: 47.1, neutropenia: 35.3, anemia: 41.2, thrombocytopenia: 47.1, asthenia: 52.8, anorexia: 35.3, nausea: 52.8, vomiting: 47.1, diarrhea: 17.6, fatigue: 23.5, abdominal pain: 23.5, skin rash: 17.6, hand–foot syndrome: 11.8, pigmentation: 5.8, Stomatitis: 5.8, lacrimation increased: 11.8, tinnitus: 5.8, ALT elevation: 17.6, AST elevation: 17.6, increased creatinine: 5.8, hyponatraemia: 5.8, hypoalbuminemia: 23.5 Control group: leucopenia: 38.90%, neutropenia: 50%, anemia: 38.90%, thrombocytopenia: 44.40%, asthenia: 44.40, anorexia:50, nausea: 50, vomiting: 38.90, diarrhea: 22.20, fatigue: 27.80, abdominal pain: 16.70, skin rash: 22.20, hand–foot syndrome: 16.70, pigmentation: 5.60, stomatitis: 11.10, lacrimation increased: 16.70, tinnitus: 11.10, ALT elevation: 22.20, AST elevation: 27.80, increased creatinine: 11.10, hyponatraemia: 11.10, hypoalbuminemia: 22.20	17/5; 7/3; NA/NA NR/13: NR/6: NA/NA
Qiao, 2019 (12)	CT; 13.7	28	13/15	5/8; 6/9	62.1 ± 10.6; 62.4 ± 14.6	<2; NM	III, IV	DC-CIK	2.8; 2; 3; day 15, 17, 19; 3 weeks	S-1	Treatment group: leucopenia:69.2, neutropenia:69.2, anemia:76.9, thrombocytopenia:61.5, asthenia:69.2 , anorexia:76.9, nausea:76.9, vomiting:53.8, diarrhea:30.8, fatigue:30.8, abdominal pain:23.1, skin rash:15.4, hand–foot syndrome:15.4, pigmentation:7.7, stomatitis:15.4, lacrimation increased:7.7, tinnitus:15.4 ALT elevation:30.8, AST elevation:38.5, increased creatinine:15.4, hyponatraemia:15.4, hypoalbuminemia: 38.5 Control group: fatigue: 46.7, abdominal pain:26.7, skin rash:20, hand–foot syndrome: 20, pigmentation: 13.3, stomatitis: 13.3, lacrimation increased:20, tinnitus:13.3, ALT elevation: 33.3, AST elevation:40, increased creatinine: 13.3, hyponatremia: 13.3, hypoalbuminemia: 33.3	NR/13; NR/6; NA/NA
Shan, 2014 (30)	RCT; NM	90	45/45	NA; NA	35.3 ± 10.4; 36.5 ± 8.7	NM; ≥60	NM	DC-CIK	10; 1; NA; NA; NA	Paclitaxel + cisplatin; NA	NA	NA/NA; NA/NA; NA/NA
Shi, 2012 (31)	RCT; median: 50.5, range: 18-82	151	74/77	43/31; 58/19	58 ± 2.1; 56 ± 1.1	<2; NM	III, IV	CIK	1; 3; 5; every second day; 8–12 weeks	5-FU (6 cycles); gastrectomy	Treatment group: Fever: 20.8, chills: 14.8, headache: 10, rash: 5.1, nausea/vomiting: 5	48.1/42.1; NA/NA; 40.4/34.1
Wang, 2017 (13)	RCT; median 48.6, range: 16.3-69.6	159	51/96	38/13;73/23	NA; NA	≤2; NM	II, III	CIK	1.2-2; 6; 8; NA; 3 weeks	5-FU + cisplatin (NA), gastrectomy	Treatment group: Fever: 15.9, bone marrow suppression: 52 Control group: bone marrow suppression: 59	NR/NR; NA/NA; NR/NR
Wu, 2015 (32)	RCT	52	28/28	NA; NA	40-65; 45-68	NM; after 85 17 75 10	II-IV	DC-CIK	NA; NA; NA; NA; NA	Gemcitabine + oxaliplatin; NA	NA	NA/NA; NA/NA; NA/NA
Xiaoliang, 2020 (35)	Retrospective; 12.5	96	48/48	29/19; 32/16	56 (median); 55 (median)	<2; NM	II, III	CIK	1; 6-8; 4; NA; 3 weeks	XELOX or FOLFOX; radical gastrectomy	Treatment group: Lymphopenia: 58.3, anemia: 33.3, thrombocytopenia: 43.8, vomiting: 62.5, hepatic insufficiency: 18.8, Diarrhea: 16.7, fever: 16.7, peripheral neuropathy: 12.5	16/14.5; NA/NA; NA/NA
Xu, 2015 (33)	RCT; 36	66	30/36	NA; NA	NA; NA	NA; NA	III-IV	CIK	30-60; 1; NA; NA; NA	Docetaxel + oxaliplatin + S1 (Gimeracil and Oteracil Porassium Capsules); NA	NA	18.89/1.78; 16.67/12.26; NA/NA
Zhao, 2013 (34)	Retrospective; 118	165	53/112	40/13; 87/25	NA; NA	NM; >60%	II, III	CIK	5; 3; 2; day 15 and 16; 4 weeks	FUP or FOLFOX4; surgery	Treatment group: Fever: 5.66	96/32; 36/23; NA;NA

CT: Clinical Trial; RCT: Randomized controlled trial; ALT: Alanine aminotransferase; AST: Aspartate aminotransferase; CIK: Cytokine-induced killer cell; 5-FU: 5-Flurouacil; NA: Not applicable; NM: Not mentioned.

Phenotypic analysis of T lymphocytes and subsets of the CIK/CIK-DC group were reported in 13 studies and were summarized in **Table 2** (**Table 2**).

**Table 2:** Phenotypic analysis of T lymphocytes and subsets of the CIK/CIK-DC group before and after treatment.

Author, year^a^	CD3				CD4				CD8				CD4/CD8				NK (CD3+, CD56+)			
	Before		After		Before		After		Before		After		Before		After		Before		After	
	Mean	SD	Mean	SD	Mean	SD	Mean	SD	Mean	SD	Mean	SD	Mean	SD	Mean	SD	Mean	SD	Mean	SD
Chen 2015 (18)	NA	NA	88.4	7.5	NA	NA	8.3	3.2	NA	NA	72	4.5	NA	NA	NA	NA	NA	NA	NA	NA
Cui 2015 (19)	NA	NA	NA	NA	NA	NA	NA	NA	NA	NA	NA	NA	NA	NA	NA	NA	8.01	9.34	85.32	14.29
Gao 2014 (20)	NA	NA	NA	NA	NA	NA	NA	NA	NA	NA	55.62	7.98	NA	NA	NA	NA	NA	NA	28.61	7.67
Jiang 2006 (23)	49.6	11.7	58.2	11.7	31.5	8.4	38.5	9.7	32.7	9.5	28.6	5.1	0.9	0.1	1.4	0.2	15.6	3.3	26.4	7
Liu 2013 (26)	57.21	9.44	66.75	9.81	29.54	7.82	38.12	8.02	26.32	6.48	19.62	5.92	1.06	0.25	1.37	0.29	18.23	6.12	26.11	6.48
Liu 2022 (22)	55.17	3.73	60.61	3.63	33.13	4.09	36.51	4.47	27.17	5.14	26.34	5.19	1.43	0.12	1.56	0.14	16.52	3.98	20.19	4.22
Lv 2015 (28)	64.7	9.8	76.4	11.5	30.6	5.5	40.5	6.6	34.7	7	26.6	5.2	0.9	0.2	1.5	0.2	9.7	2.9	17.6	3.8
Mu 2016 (29)	61.3	10.6	65.2	11.5	30.3	6.3	36.1	7.5	29.6	6.1	28.2	6.3	1.03	0.21	1.33	0.28	9.6	2.6	14.6	3.3
Qiao 2019 (12)	40.35	7.7	83.72	6.2	40.22	5.13	46.86	6.79	25.65	3.65	25.38	4.51	NA	NA	NA	NA	17.71	3.42	15.83	3.77
Shi 2012 (31)	50.8	8.5	63.6	11.3	26.5	6.1	36	6.6	NA	NA	NA	NA	0.9	0.4	1.4	0.3	NA	NA	NA	NA
Wang 2017 (13)	9.38	3.66	34.4	11.31	NA	NA	NA	NA	NA	NA	NA	NA	NA	NA	NA	NA	11.94	3.62	80.17	13.33
Xiaoliang 2020 (35)	76.12	8.3	78.4	10.07	35.13	5.14	38.34	9.83	32.76	5.25	36.82	7.53	NA	NA	NA	NA	6.39	3.28	7.05	4.22
Zhao 2013 (34)	48.95	6.89	80.7	9.21	29.23	4.87	42.75	7.72	19.25	5.64	36.1	9.27	NA	NA	NA	NA	15.1	4.74	30.21	7.53

^a^ Cui 2014, Fan 2012, Jiang 2008, Jiang 2010, Li 2017, Ma 2023, Shan 2014, Wu 2015, and Xu 2015 did not provide data on cell subtypes before and after CIK/DC-CIK therapy in the treatment group.

### 3.2 Quality assessment

The assessment of the risk of bias for each included study is shown in **Supplementary Figure 1A**. Overall, the quality of the studies ranged from unclear to high. 9 studies were judged as high risk, and the remaining 13 studies did not provide a clear description of the overall risk of bias. **Supplementary Figure 1B** demonstrates each risk of bias item presented as percentages across all included studies. In total, for >50% of studies, random sequence generation, blinding of participants and personnel, blinding of outcome assessment, incomplete outcome data, and other bias showed low risk of bias, while most studies for allocation concealment and selective reporting had unclear or high risk of bias.

### 3.3 Results of meta-analysis

#### 3.3.1 Overall survival rates

**Figure 2:**
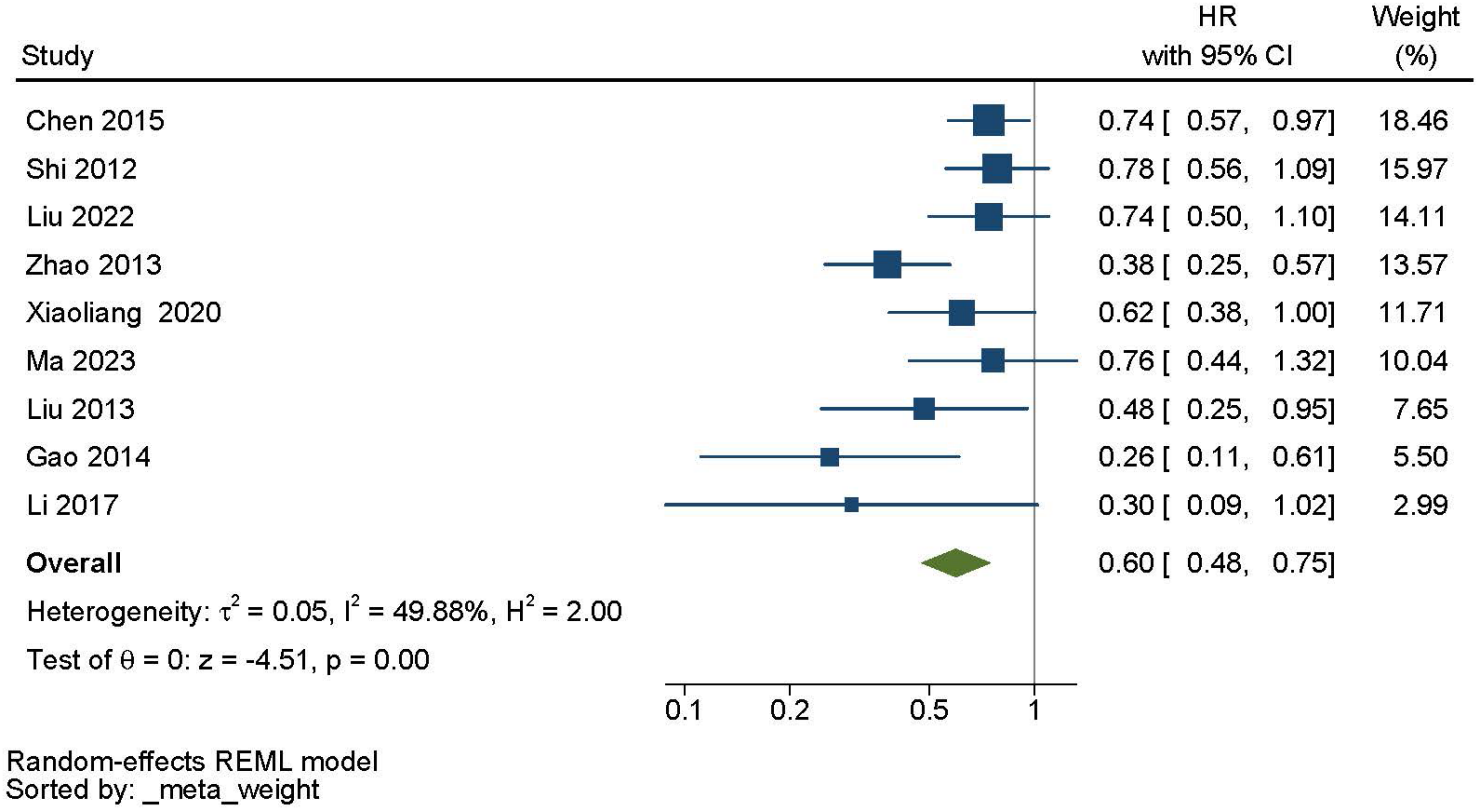
Forest plot of overall survival (OS) comparing cytokine-induced killer cell (CIK) or dendritic cell-cytokine-induced killer cell (DC-CIK) therapy plus chemotherapy versus chemotherapy alone in patients with gastric cancer.

Across 9 studies including 1,047 patients, the random-effects (REML) meta-analysis of overall survival showed that CIK/DC-CIK therapy was associated with a significantly reduced risk of death compared with chemotherapy (pooled HR 0.60, 95% CI 0.48–0.75; p < 0.001). Most included studies reported hazard ratios <1 in favor of CIK/DC-CIK therapy, although a few individual estimates crossed the null. Between-study heterogeneity was moderate (I² = 49.9%), indicating some variability in effect size across studies. Overall, these results suggest that CIK/DC-CIK therapy confers a meaningful overall survival advantage over chemotherapy (**Figure 2**).

#### 3.3.2 Progression-free survival rates

**Figure 3:**
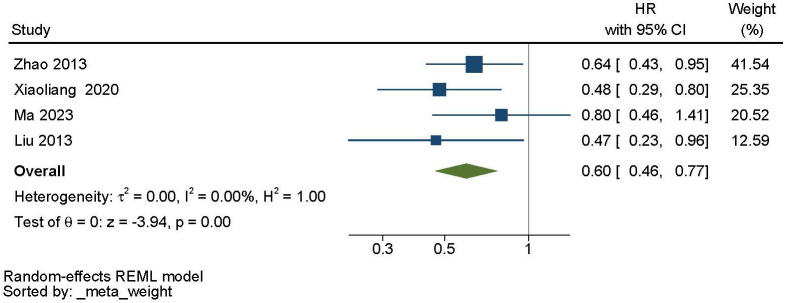
Forest plot of progression-free survival (PFS) comparing CIK/DC-CIK plus chemotherapy versus chemotherapy alone in patients with gastric cancer.

Across 4 studies including 418 patients, the random-effects (REML) meta-analysis of progression-free survival (PFS) showed a statistically significant benefit of CIK therapy compared with chemotherapy, with a pooled HR of 0.60 (95% CI 0.46–0.77; *p* < 0.001). Most individual studies reported hazard ratios below unity in favor of CIK/DC-CIK therapy, although one study showed a non-significant effect despite a consistent direction of benefit. No between-study heterogeneity was observed (*I*² = 0.0%), indicating consistent effects across included studies. Overall, these findings suggest that CIK/DC-CIK therapy provides a meaningful improvement in PFS relative to chemotherapy (**Figure 3**).

#### 3.3.3 Disease-free survival rates

**Figure 4:**
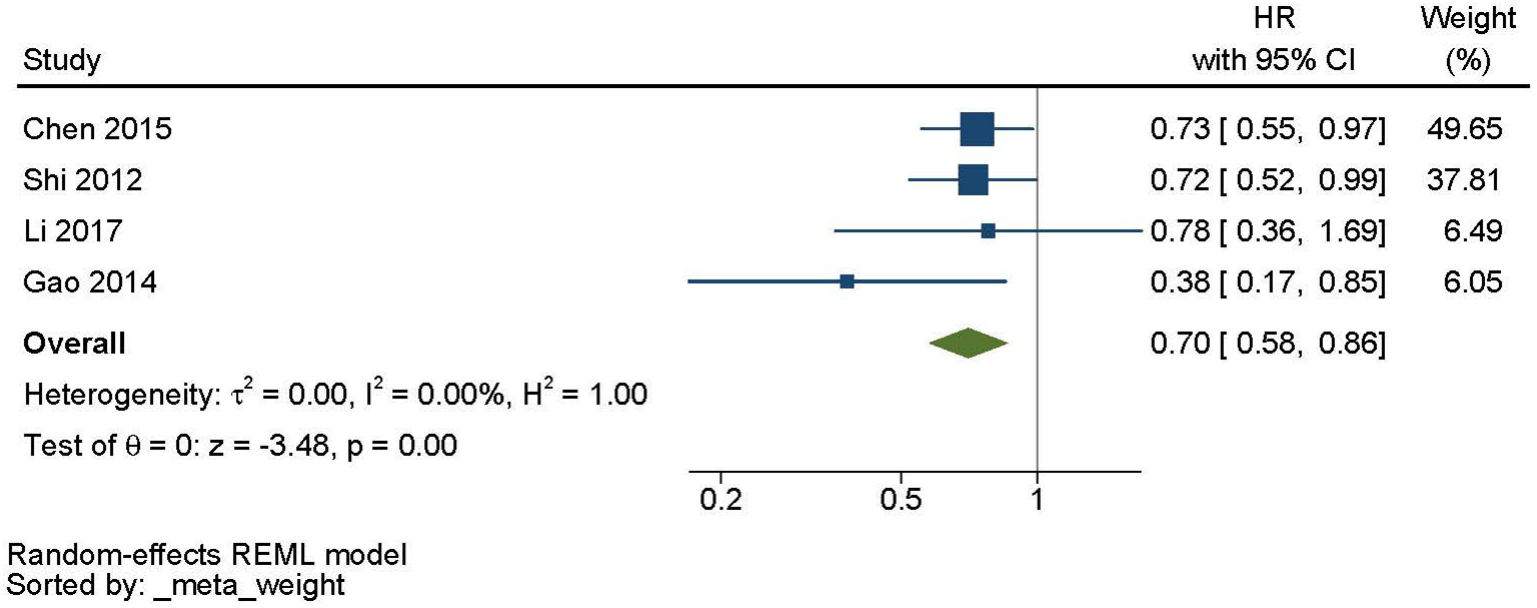
Forest plot of disease-free survival (DFS) comparing CIK/DC-CIK plus chemotherapy versus chemotherapy alone in patients with gastric cancer.

Across 4 studies including 523 patients, the random-effects (REML) meta-analysis of disease-free survival (DFS) demonstrated a statistically significant benefit of CIK/DC-CIK therapy compared with chemotherapy, with a pooled HR of 0.70 (95% CI 0.58–0.86; *p* < 0.001). Individual study estimates consistently favored CIK/DC-CIK therapy, with three of four studies reporting statistically significant reductions in the risk of disease recurrence, while one study showed a directionally similar but non-significant effect. There was no observed heterogeneity among the included studies (*I*² = 0.0%), indicating highly consistent effects across cohorts. Overall, these findings suggest that CIK/DC-CIK therapy provides a meaningful improvement in disease-free survival relative to chemotherapy (**Figure 4**).

#### 3.3.4 Objective response rate

**Figure 5:**
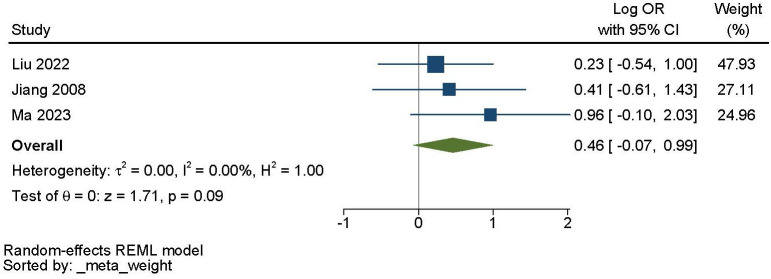
Forest plot of objective response rate (ORR) comparing CIK/DC-CIK plus chemotherapy versus chemotherapy alone in patients with gastric cancer.

From three studies (225 patients), the meta-analysis showed a non-significant trend toward a higher ORR with CIK/DC-CIK therapy compared with control (pooled log OR 0.46, 95% CI −0.07 to 0.99;, p=0.09), with no observed heterogeneity (I²=0%) (**Figure 5**).

#### 3.3.5 Disease control rate

**Figure 6:**
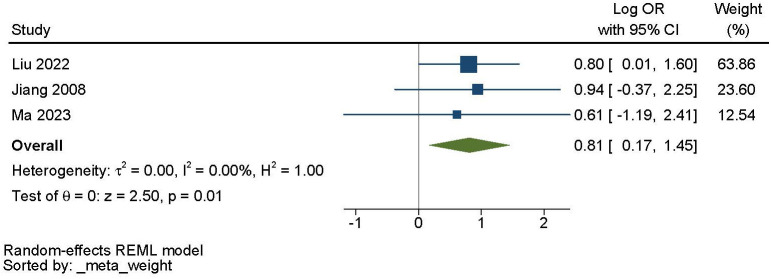
Forest plot of disease control rate (DCR) comparing CIK/DC-CIK plus chemotherapy versus chemotherapy alone in patients with gastric cancer.

Based on 3 studies (225 patients), CIK/DC-CIK therapy was associated with a significantly higher DCR than control, with a pooled log OR of 0.81 (95% CI 0.17 to 1.45; z=2.50, p=0.01). No between-study heterogeneity was detected (I²=0.00%) (**Figure 6**).

#### 3.3.6 Subgroup analysis and meta-regression analysis

Subgroup analysis comparing CIK versus DC-CIK showed no evidence that the overall survival (OS) effect differed by product type (test for group differences; p=0.15; **Supplementary Figures 5**). The pooled estimate was statistically significant for the CIK subgroup, whereas the DC-CIK subgroup showed a non-significant trend, but the between-subgroup difference was not significant.

In the subgroup analysis by study design, both RCTs and non-RCTs showed an overall survival benefit with CIK/DC-CIK therapy compared with control, and the effect did not differ significantly between designs (test for group differences p=0.32). In the RCT subgroup (5 studies), the pooled effect indicated a significantly reduced risk of death (HR = 0.68, 95% CI 0.54–0.84; p<0.001) with low heterogeneity (I²=4.00%). In the non-RCT subgroup (4 studies), the pooled estimate was similarly significant (HR = 0.54, 95% CI 0.37–0.79; p<0.001), although heterogeneity was substantial (I²=62.65%) (**Supplementary Figure 6**).

In meta‐regression exploring potential sources of heterogeneity in the primary survival analysis, immune‐phenotype changes emerged as significant moderators of the treatment effect. Specifically, greater post-treatment increases in NK cells, CD4+ T cells, and CD3+ T cells were each associated with a more favorable survival effect. NK change showed a significant inverse association with the effect size (β = −0.043, SE = 0.02; p = 0.032; 95% CI −0.0830 to −0.0037; 4 studies). Similarly, CD4 change (β = −0.0627, SE = 0.0253; p = 0.013; 95% CI −0.1124 to −0.0131; 5 studies) and CD3 change (β = −0.0226, SE = 0.00915; p = 0.014; 95% CI −0.0405 to −0.00465; 5 studies) were significant, and each model explained a large proportion of between-study variability with minimal residual heterogeneity. These findings suggest that studies showing stronger immune activation (higher CD3/CD4/NK increases) tended to report larger survival benefits with CIK/DC-CIK therapy. However, because these regressions were based on a small number of studies (n = 4–5), the results should be interpreted cautiously. Other prespecified moderators (CIK dosage, gender ratio, mean age, baseline metastasis percentage, CD8 change, and CD4/CD8 change) were not consistently significant, and subgroup comparisons by CIK vs. DC-CIK and RCT vs. non-RCT did not demonstrate clear effect modification.

#### 3.3.7 Publication bias

Because OS had the largest evidence base, we assessed potential publication bias using funnel-plot methods (**Supplementary Figure 8**). Visual inspection showed no marked funnel-plot asymmetry (**Supplementary Figure 7A**). However, small-study effects were suggested by formal tests (Begg’s p=0.03; Egger’s p=0.02). To further examine robustness, we applied the trim-and-fill procedure (**Supplementary Figure 7B**), which imputed four potentially missing studies; the adjusted pooled effect remained statistically significant and in the same direction (trim-and-fill log HR equivalent: HR 0.736, 95% CI 0.645–0.839), indicating that the overall conclusion was not materially changed after accounting for potential publication bias.

#### 3.3.8 Sensitivity analysis

Again, OS (as the outcome with the largest number of included studies) was chosen to test the sensitivity analysis, ensuring the stability of the results by sequentially removing each study. The removal of any single study did not change the overall statistical results, indicating that the results of this study were statistically robust (**Supplementary Figure 8**).

#### 3.3.9 Certainty of evidence using GRADE assessment

Using the GRADE approach, the certainty of evidence for the effect of CIK/DC-CIK plus chemotherapy on overall survival was judged to be low. Although the pooled analysis showed improved OS (HR 0.60, 95% CI 0.48–0.75; p < 0.001), we downgraded the certainty for several reasons. First, we downgraded for risk of bias, because many included trials had methodological limitations and/or incomplete reporting of key safeguards (e.g., allocation concealment, blinding, and selective reporting), raising concern that effect estimates may be exaggerated. Second, we downgraded for indirectness, because all included studies were conducted in China, which may limit generalizability to other healthcare settings with different baseline regimens, staging work-up, and supportive care. Third, we downgraded for publication bias, as formal testing suggested small-study effects (Begg’s p=0.03; Egger’s p=0.02), and trim-and-fill imputed four potentially missing studies; while the adjusted estimate remained statistically significant (HR 0.736, 95% CI 0.645–0.839), the attenuation supports concern for selective publication. We also downgraded for inconsistency due to moderate heterogeneity in the pooled OS estimate (I² = 49.9%). We did not downgrade for imprecision, because the confidence interval excluded the null and the estimate was reasonably precise. Overall, these considerations support low-certainty evidence that adding CIK/DC-CIK to chemotherapy improves OS.

## 4 Discussion

GC remains a leading cause of cancer-related mortality worldwide, especially in East Asian populations (36). Despite advances in surgical techniques and chemotherapeutic regimens, the prognosis for advanced or recurrent GC remains poor (37). Conventional treatment options provide limited long-term survival, prompting the exploration of novel therapeutic approaches, including immunotherapy. CIK cells, alone or combined with DC, represent a promising form of adoptive cell therapy due to their potent cytotoxicity and non-MHC-restricted tumor-killing activity (38–40).

Considering the results of 13 studies, this meta-analysis reveals that CIK/DC-CIK therapy significantly improves OS, PFS, DFS, and DCR in patients with GC compared to standard treatments. Notably, the pooled analysis of OS across 13 studies showed an HR of 0.60, 95% CI 0.48–0.75, with moderate heterogeneity (I² = 49.9%), indicating a consistent benefit. Similarly, sustained improvements in PFS and DFS across multiple time points suggest that CIK/DC-CIK therapy may delay both disease progression and recurrence. Our results confirmed findings from previous systematic reviews and meta-analyses published previously. In 2016, Shen et al. published a systematic review and meta-analysis including 9 studies. The pooled analysis indicated that patients in the CIK/DC-CIK group had significantly better OS compared to those in the control group, with an OR of 2.64 (95% CI: 2.20-3.18). Furthermore, the treatment was associated with a notable improvement in PFS, yielding an OR of 3.01 (95% CI: 2.15–4.22). However, no information regarding DFS was provided in this review (41). Again, in 2017, Wang et al. included the same number of studies (9 studies) in their systematic review. In this study, in comparison to the control group, the hazard ratio (HR) for OS was 0.71 (95% CI: 0.59–0.85), indicating a significant survival benefit. For DFS, the HR was 0.66 (95% CI: 0.54–0.79). The risk ratios (RR) for 3-year and 5-year OS were 1.29 (95% CI: 1.15–1.46) and 1.73 (95% CI: 1.36–2.19), respectively. Likewise, the RR for 3-year and 5-year DFS were 1.40 (95% CI: 1.19–1.65) and 2.10 (95% CI: 1.53–2.87), respectively. However, they did not include data on PFS, ORR, and DCR (42). A recent meta-analysis by Du et al., which was published in 2020, also included 9 studies. Based on this systematic review, patients receiving chemotherapy in combination with CIK or DC–CIK cell therapy demonstrated significant improvements in OS (RR = 1.84; 95% CI: 1.41–2.40) and PFS (RR = 1.99; 95% CI: 1.52–2.60) (43). This study did not perform an analysis on the DFS outcome. Moreover, all of these meta-analyses merely focused on some specific time-points, but we extracted data on various time points (i.e. 6-month, 1-year,18-month, 2-year, 3-year, 4-year, and 5-year) to specify the change in primary outcomes (OS, PFS and DFS) and across time and ensure the long-term efficacy.

While data on ORR were limitedly reported in previous meta-analyses (41, 43), our updated analysis included three studies (n=225) and showed only a non-significant trend toward improved ORR with CIK/DC-CIK compared with control (pooled log OR 0.46, 95% CI −0.07 to 0.99; p=0.09). In contrast, for DCR, although Shen et al. extracted data from only one study (41), our pooled analysis of three studies (n=225) demonstrated a significant improvement in disease control with CIK/DC-CIK (pooled log OR 0.81, 95% CI 0.17 to 1.45; p=0.01). Overall, these findings suggest that, based on the currently eligible evidence, CIK/DC-CIK may have a clearer impact on disease control than on objective response, while highlighting the need for additional well-reported trials to better define response-related outcomes.

Importantly, our subgroup and sensitivity analyses lend robustness to the core findings. The therapeutic benefit was consistent regardless of study design (RCT vs. non-RCT), and heterogeneity was minimal or explainable in most endpoints. The advantage persisted across multiple follow-up intervals, especially at the 4- and 5-year timepoints, further supporting the long-term potential of adoptive immunotherapy in GC. Subgroup analysis based on therapy type (CIK vs. DC-CIK) revealed no statistically significant difference in survival outcomes, although a trend favoring DC-CIK for PFS was observed. This aligns with prior findings in other cancers, such as colorectal cancer, where co-culturing CIK cells with DCs enhances their cytolytic potential by improving antigen presentation and T-cell activation (44). However, our results suggest that while DC augmentation may improve short- to medium-term immune responses (e.g., PFS), it does not translate to a consistent OS advantage. Future trials should explore whether other combinations, such as CIK with checkpoint inhibitors or CAR-T constructs or tumor infiltrating lymphocytes, can enhance survival further (45–47). Meta-regression identified gender ratio as a significant source of heterogeneity, with female patients showing better PFS outcomes. This intriguing finding suggests potential immunological or hormonal factors influencing therapy efficacy, warranting further investigation. Moreover, differences in DFS heterogeneity between RCTs and non-RCTs underscore the importance of standardized study designs in generating reproducible immunotherapy data.

Compared to other types of cancers, where similar meta-analyses have shown promise for CIK therapy (26, 44, 48–50), the current analysis confirms the broad applicability of CIK/DC-CIK therapy in solid tumors, including GC. The consistency across multiple time points and clinical outcomes underscores the clinical relevance of these adoptive cellular therapies beyond hematologic malignancies.

Nonetheless, this analysis has limitations. All included studies were conducted in China, limiting external validity due to this geographic concentration. The effectiveness and safety of CIK/DC-CIK therapy may be influenced by differences in patient demographics, gastric cancer epidemiology, tumor biology, stage distribution, prior treatments, chemotherapy backbones, surgical practices, supportive care, and follow-up protocols. In addition, preparation, characterization, dosing, and regulatory oversight of adoptive cellular therapies may vary across institutions and countries. Therefore, the present findings should be interpreted as evidence primarily applicable to Chinese clinical settings. Validation in diverse populations and healthcare systems through well-designed international multicenter randomized trials is needed before the results can be generalized broadly. Accordingly, the results should be interpreted with caution and may not be readily generalizable to other populations or healthcare settings.

Variability in CIK preparation protocols, such as cytokine concentrations, culture durations, and patient selection criteria, could also contribute to outcome heterogeneity. While basic standards for CIK production exist, the lack of uniform cell characterization prior to transfusion hampers direct comparisons across trials.

Furthermore, data on immune-related adverse events and quality of life metrics were sparse, limiting a comprehensive safety assessment. Safety reporting was incomplete and non-standardized across the included trials. Although fever was the most frequently reported infusion-related event and no fatal adverse events were reported, most studies did not provide extractable arm-level counts for grade ≥3 adverse events, serious adverse events, treatment discontinuations, or adverse events graded according to standardized criteria such as Common Terminology Criteria for Adverse Events (CTCAE). Therefore, the safety findings should be interpreted cautiously. The absence of consistently reported severe adverse events should not be interpreted as definitive evidence that CIK/DC-CIK therapy does not increase severe toxicity. Rather, the available evidence is insufficient to support a robust comparative safety conclusion. Future trials should prospectively collect and report adverse events using standardized grading systems, including grade ≥3 toxicities, serious adverse events, immune-related adverse events, and treatment discontinuations.

In addition, the overall certainty of evidence was limited by methodological quality. Risk-of-bias assessment identified nine studies at high risk of bias, and we therefore excluded these trials from the quantitative synthesis, resulting in a reduced evidence base for pooled estimates. Although this approach improves internal validity, it may also reduce precision and highlights the reliance of the literature on incompletely reported study methods. Consistent with this, the certainty of evidence for the primary outcome (OS) was rated low using the GRADE approach, with downgrading driven by concerns regarding risk of bias (incomplete reporting of key safeguards such as allocation concealment, blinding, and selective reporting), inconsistency (moderate heterogeneity in the pooled OS estimate), indirectness (all studies conducted in China, limiting generalizability), and publication bias (evidence of small-study effects and attenuation after trim-and-fill). Collectively, these limitations indicate that the magnitude of benefit may be overestimated and reinforce the need for adequately powered, well-reported, multicenter RCTs.

Despite these limitations, our findings support assessing integration of CIK/DC-CIK therapy into standard GC treatment regimens, especially for patients with unresectable, recurrent, or metastatic disease. The therapy was generally well-tolerated, with low heterogeneity in ORR and DCR outcomes, suggesting a favorable risk-benefit profile. However, because severe adverse events were inconsistently reported and the certainty of evidence was low, these findings should be considered exploratory rather than sufficient to support broad clinical adoption.

## 5 Conclusion

CIK/DC-CIK therapy combined with chemotherapy was associated with improved survival outcomes in patients with gastric cancer; however, the strength and generalizability of the evidence remain limited. The available evidence is derived entirely from studies conducted in China, with variable study designs, reporting quality, and moderate heterogeneity for overall survival. In addition, the certainty of evidence for OS was judged to be low by GRADE, and key safety outcomes (e.g., grade ≥3 adverse events) were inconsistently reported. Therefore, these findings should be interpreted cautiously and may not be generalizable to other populations or healthcare settings. Well-designed, adequately powered, international multicenter RCTs with standardized protocols, transparent risk-of-bias safeguards, and comprehensive harms reporting are needed before broad clinical adoption can be recommended.

## Data Availability

The original contributions presented in the study are included in the article/**Supplementary Material**. Further inquiries can be directed to the corresponding authors.
