# Supplementary material for: Safety and efficacy of cytokine-induced killer cells for gastric cancer: a systematic review and meta-analysis
Source: Front Oncol. 2026 Jun 8;16:1834073. doi: 10.3389/fonc.2026.1834073 (PMC13283801; doi:10.3389/fonc.2026.1834073)
Supplement: Supplementary file 1 [file DataSheet1.docx]

| Table S1- Detailed search strategy and results in each database | | |
| --- | --- | --- |
| Database | Search strategy | Results (September 2025) |
| PubMed | (((((((((Gastric Carcinoma[Title/Abstract]) OR (Gastric Cancer[Title/Abstract])) OR (Gastric Tumor[Title/Abstract])) OR (Gastric Neoplasm[Title/Abstract])) OR (Stomach Carcinoma[Title/Abstract])) OR (Stomach Cancer[Title/Abstract])) OR (Stomach Tumor[Title/Abstract])) OR (Stomach Neoplasm[Title/Abstract])) OR ("Stomach Neoplasms"[Mesh])) AND ((((((Cytokine Induced Killer Cell[Title/Abstract]) OR (Cytokine-Induced Killer Cell[Title/Abstract])) OR (CIK Cell[Title/Abstract])) OR (Lymphocyte-Activated Killer Cell[Title/Abstract])) OR (Lymphocyte Activated Killer Cell[Title/Abstract])) OR ("Cytokine-Induced Killer Cells"[Mesh])) | 254 |
| Scopus | ( ( TITLE-ABS-KEY ( gastric AND carcinoma ) OR TITLE-ABS-KEY ( gastric AND cancer ) OR TITLE-ABS-KEY ( gastric AND tumor ) OR TITLE-ABS-KEY ( gastric AND neoplasm ) OR TITLE-ABS-KEY ( stomach AND carcinoma ) OR TITLE-ABS-KEY ( stomach AND cancer ) OR TITLE-ABS-KEY ( stomach AND tumor ) OR TITLE-ABS-KEY ( stomach AND neoplasm ) ) ) AND ( ( TITLE-ABS-KEY ( cytokine AND induced AND killer AND cell ) OR TITLE-ABS-KEY ( cytokine-induced AND killer AND cell ) OR TITLE-ABS-KEY ( cik AND cell ) OR TITLE-ABS-KEY ( lymphocyte-activated AND killer AND cell ) OR TITLE-ABS-KEY ( lymphocyte AND activated AND killer AND cell ) ) ) | 521 |
| Web of Science | 1:  Gastric Carcinoma (Topic) or Gastric Cancer (Topic) or Gastric Tumor (Topic) or Gastric Neoplasm (Topic) or Stomach Carcinoma (Topic) or Stomach Cancer (Topic) or Stomach Tumor (Topic) or Stomach Neoplasm  2:  Cytokine Induced Killer Cell (Topic) or Cytokine-Induced Killer Cell (Topic) or CIK Cell (Topic) or Lymphocyte-Activated Killer Cell (Topic) or Lymphocyte Activated Killer Cell (Topic)  #1 AND #2 | 244 |
| Total results | | 1019 |
| Duplicates | | 209 |
| Total results without duplicates | | 810 |


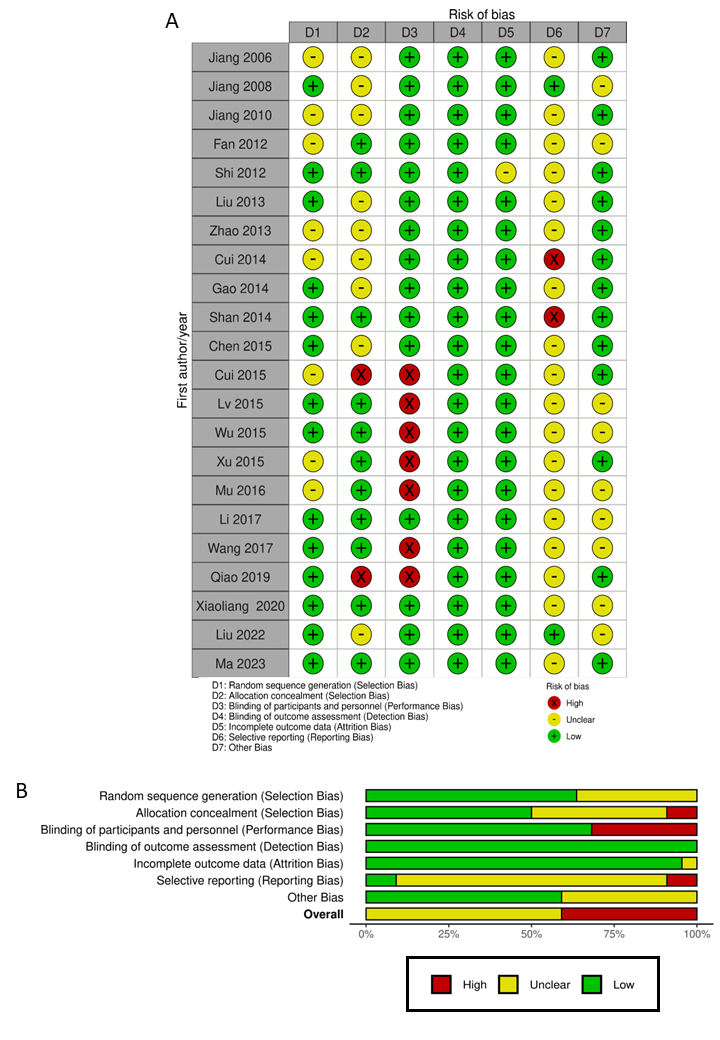


**Figure S1- (A) Risk of bias assessment summary for included studies. (B) Risk of bias graph indicating** **each risk of bias item presented as percentages across all included studies.**

**
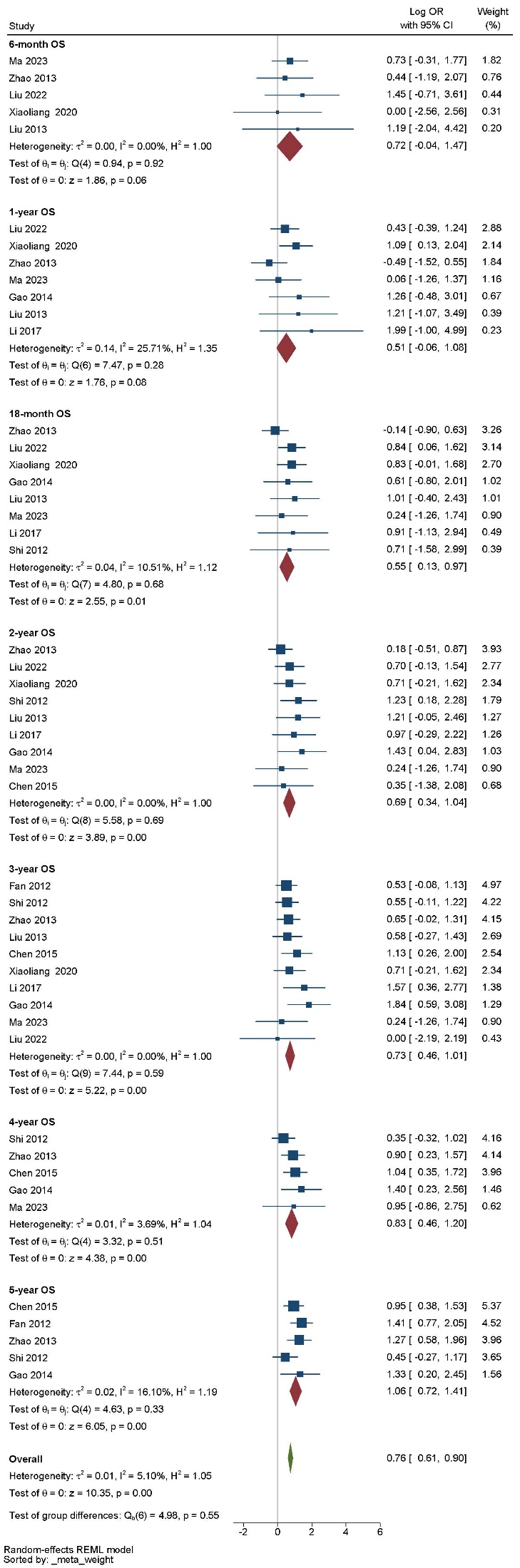
**

**Figure S2- Effect of Cytokine-Induced Killer Cells on OS in patients with gastric cancer across different timepoints during follow-up**, Ten studies involving 1212 patients contributed to the meta-analysis of overall survival (OS) across follow-up time points (Figure S2). Overall, CIK/DC-CIK therapy was associated with significantly better OS than control, with a pooled log OR of 0.76 (95% CI 0.61 to 0.90; p<0.001), corresponding to an OR of 2.14 (95% CI 1.84 to 2.46). Between-study heterogeneity was low (I²=5.1%). In total, 5, 7, 8, 9, 10, 5, and 5 studies contributed data to the 6-month, 1-year, 18-month, 2-year, 3-year, 4-year, and 5-year OS analyses, respectively. The pooled results showed a generally consistent advantage for CIK/DC-CIK therapy, with statistical significance emerging from mid-term follow-up onward. At 6 months, OS showed a borderline, non-significant trend (log OR 0.72, 95% CI −0.04 to 1.47; p=0.06; I²=0%). At 1 year, the effect remained non-significant (log OR 0.51, 95% CI −0.06 to 1.08; p=0.08; I²=25.71%). From 18 months onward, pooled effects favored CIK/DC-CIK therapy and were statistically significant: 18 months (log OR 0.55, 95% CI 0.13 to 0.97; p=0.01; I²=10.51%), 2 years (log OR 0.69, 95% CI 0.34 to 1.04; p<0.001; I²=0%), 3 years (log OR 0.73, 95% CI 0.46 to 1.01; p<0.001; I²=0%), 4 years (log OR 0.83, 95% CI 0.46 to 1.20; p<0.001; I²=3.69%), and 5 years (log OR 1.06, 95% CI 0.72 to 1.41; p<0.001; I²=16.10%). There was no evidence that the treatment effect differed across follow-up intervals (test for subgroup differences p=0.55).

**
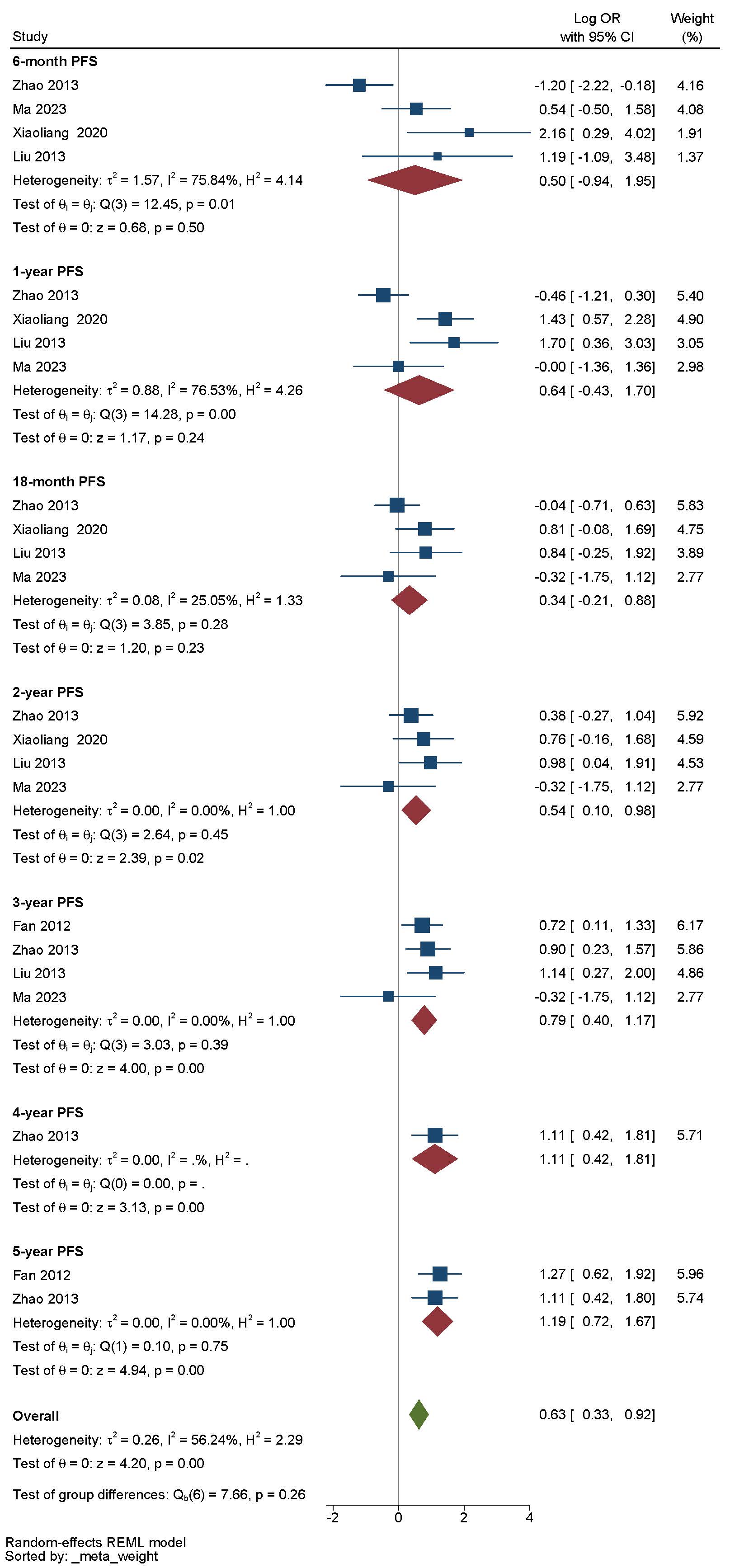
**

**Figure S3- Effect of Cytokine-Induced Killer Cells on PFS in patients with gastric cancer across different timepoints during follow-up**, For PFS, five studies involving 613 patients contributed data to the meta-analysis (Figure 3). Overall, CIK/DC-CIK therapy was associated with significantly improved PFS compared with control, with a pooled log OR of 0.63 (95% CI 0.33 to 0.92; p<0.001). Between-study heterogeneity was moderate (I²=56.24%). Across follow-up intervals, 4, 4, 4, 4, 4, 1, and 2 studies contributed to the 6-month, 1-year, 18-month, 2-year, 3-year, 4-year, and 5-year PFS rate meta-analyses, respectively. At 6 months, there was a non-significant trend favoring CIK/DC-CIK (log OR 0.50, 95% CI −0.94 to 1.95; p=0.50) with substantial heterogeneity (I²=75.84%). Similarly, at 1 year (log OR 0.64, 95% CI −0.43 to 1.70; p=0.24; I²=76.53%) and 18 months (log OR 0.34, 95% CI −0.21 to 0.88; p=0.23; I²=25.05%), effects favored therapy but did not reach statistical significance. From 2 years onward, the pooled effects were statistically significant and consistently favored CIK/DC-CIK: 2 years (log OR 0.54, 95% CI 0.10 to 0.98; p=0.02; I²=0%), 3 years (log OR 0.79, 95% CI 0.40 to 1.17; p<0.001; I²=0%), 4 years (log OR 1.11, 95% CI 0.42 to 1.81; p<0.001; single study), and 5 years (log OR 1.19, 95% CI 0.72 to 1.67; p<0.001; I²=0%). There was no evidence that the treatment effect differed by follow-up interval (test for group differences p=0.26).


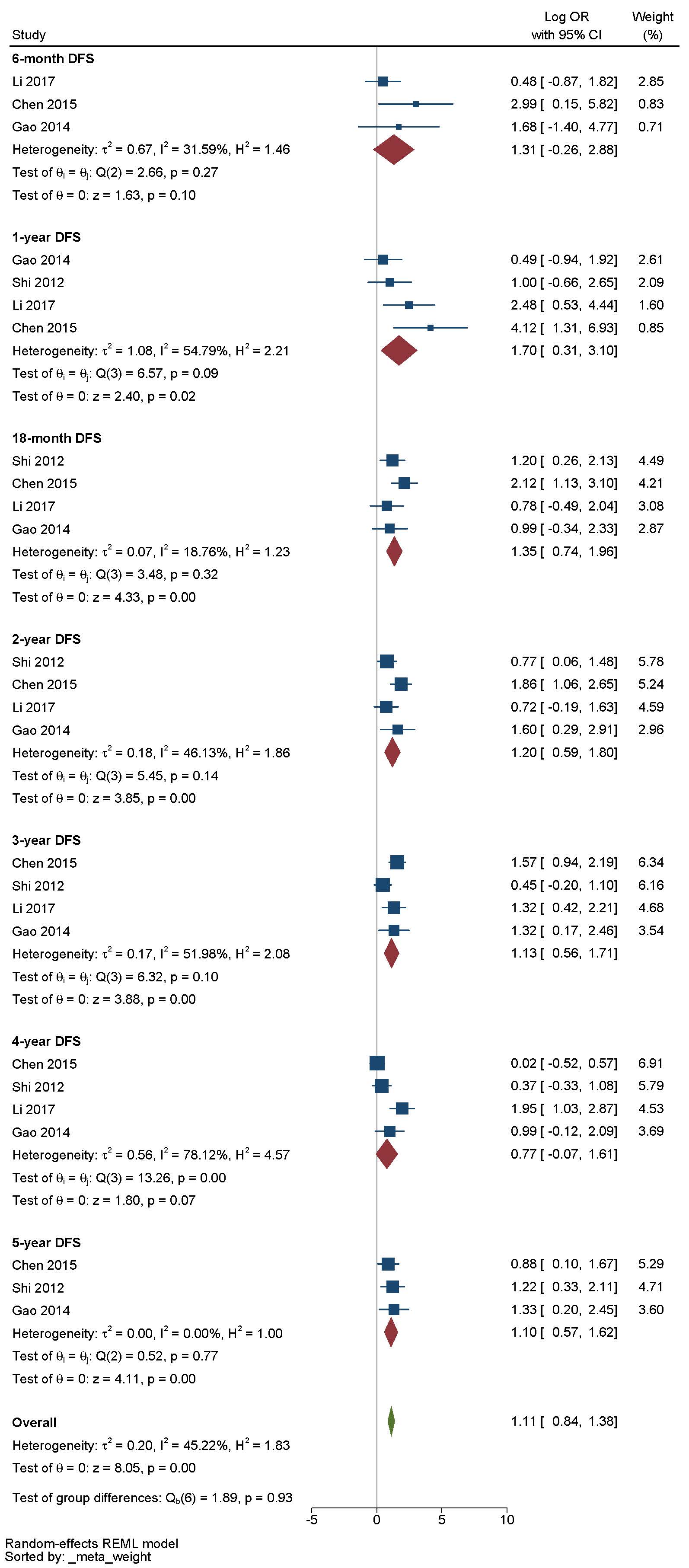


**Figure S4- Effect of Cytokine-Induced Killer Cells on DFS in patients with gastric cancer across different timepoints during follow-up**, For DFS, four studies involving 523 patients contributed data to the meta-analysis (Figure 4). Overall, CIK/DC-CIK therapy was associated with significantly improved DFS compared with control, with a pooled log OR of 1.11 (95% CI 0.84 to 1.38; p<0.001), corresponding to an OR of 3.03 (95% CI 2.32 to 3.98). Between-study heterogeneity was moderate (I²=45.22%). Across follow-up intervals, 3, 4, 4, 4, 4, 4, and 3 studies contributed to the 6-month, 1-year, 18-month, 2-year, 3-year, 4-year, and 5-year DFS rate meta-analyses, respectively. At 6 months, there was a non-significant trend favoring CIK/DC-CIK (log OR 1.31, 95% CI −0.26 to 2.87; p=0.10; I²=31.59%). At 1 year, the pooled effect was statistically significant (log OR 1.70, 95% CI 0.31 to 3.10; p=0.02) with moderate heterogeneity (I²=54.79%). Significant benefits persisted at 18 months (log OR 1.35, 95% CI 0.74 to 1.96; p<0.001; I²=18.76%), 2 years (log OR 1.20, 95% CI 0.59 to 1.80; p<0.001; I²=46.13%), 3 years (log OR 1.13, 95% CI 0.56 to 1.71; p<0.001; I²=51.98%), and 5 years (log OR 1.10, 95% CI 0.57 to 1.62; p<0.001; I²=0%). At 4 years, the pooled estimate favored CIK/DC-CIK but did not reach statistical significance (log OR 0.77, 95% CI −0.07 to 1.61; p=0.07) and heterogeneity was high (I²=78.12%), warranting cautious interpretation. There was no evidence that treatment effects differed by follow-up interval (test for group differences p=0.93).


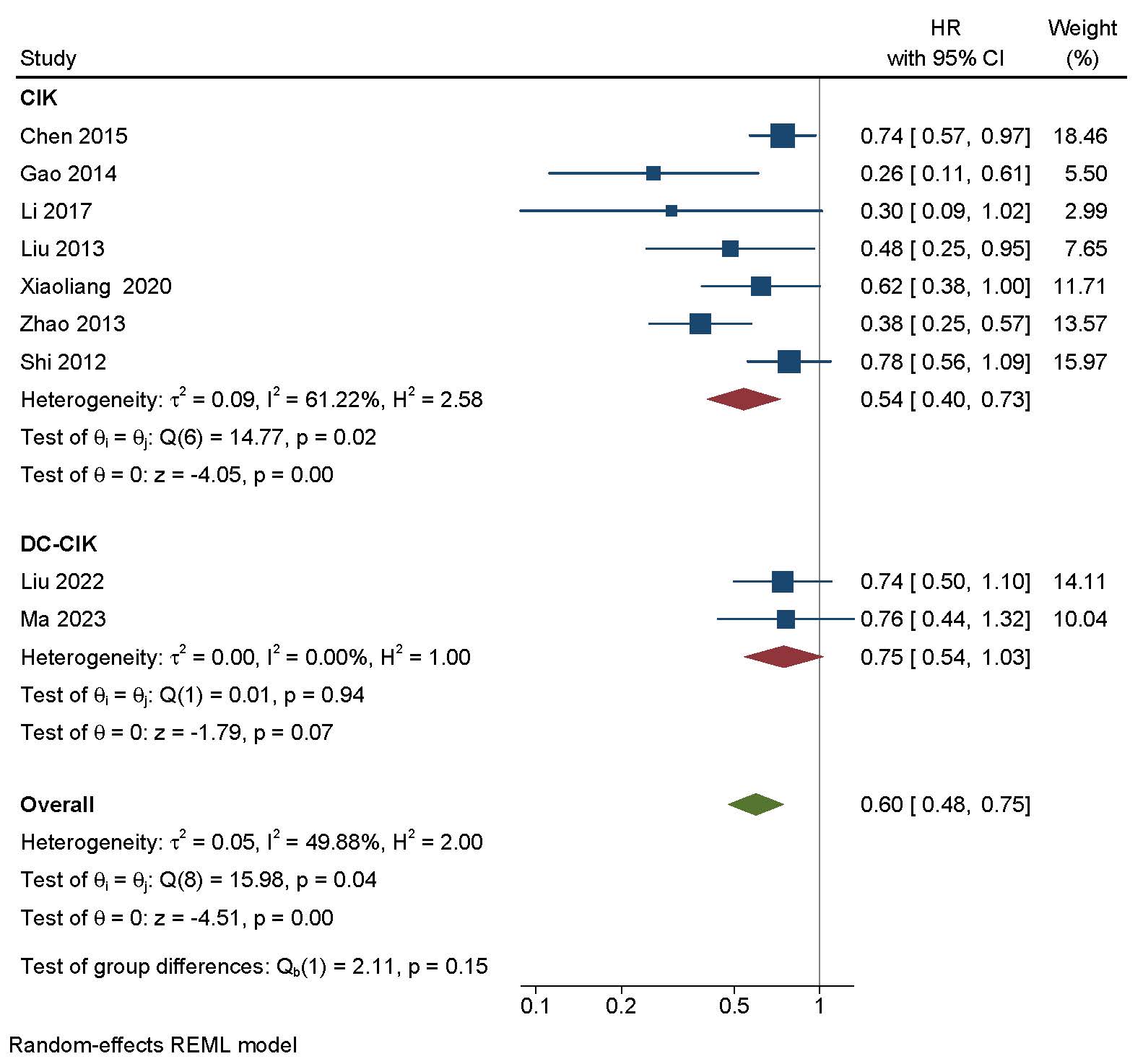


**Figure S5- Subgroup analysis of overall survival (OS) comparing cytokine-induced killer cell (CIK) vs dendritic cell-cytokine-induced killer cell (DC-CIK). There is no significant difference between CIK and DC-CIK regarding OS, however, there is still considerable heterogeneity among the studies on CIK cells (I^2^ = 61.22%).**


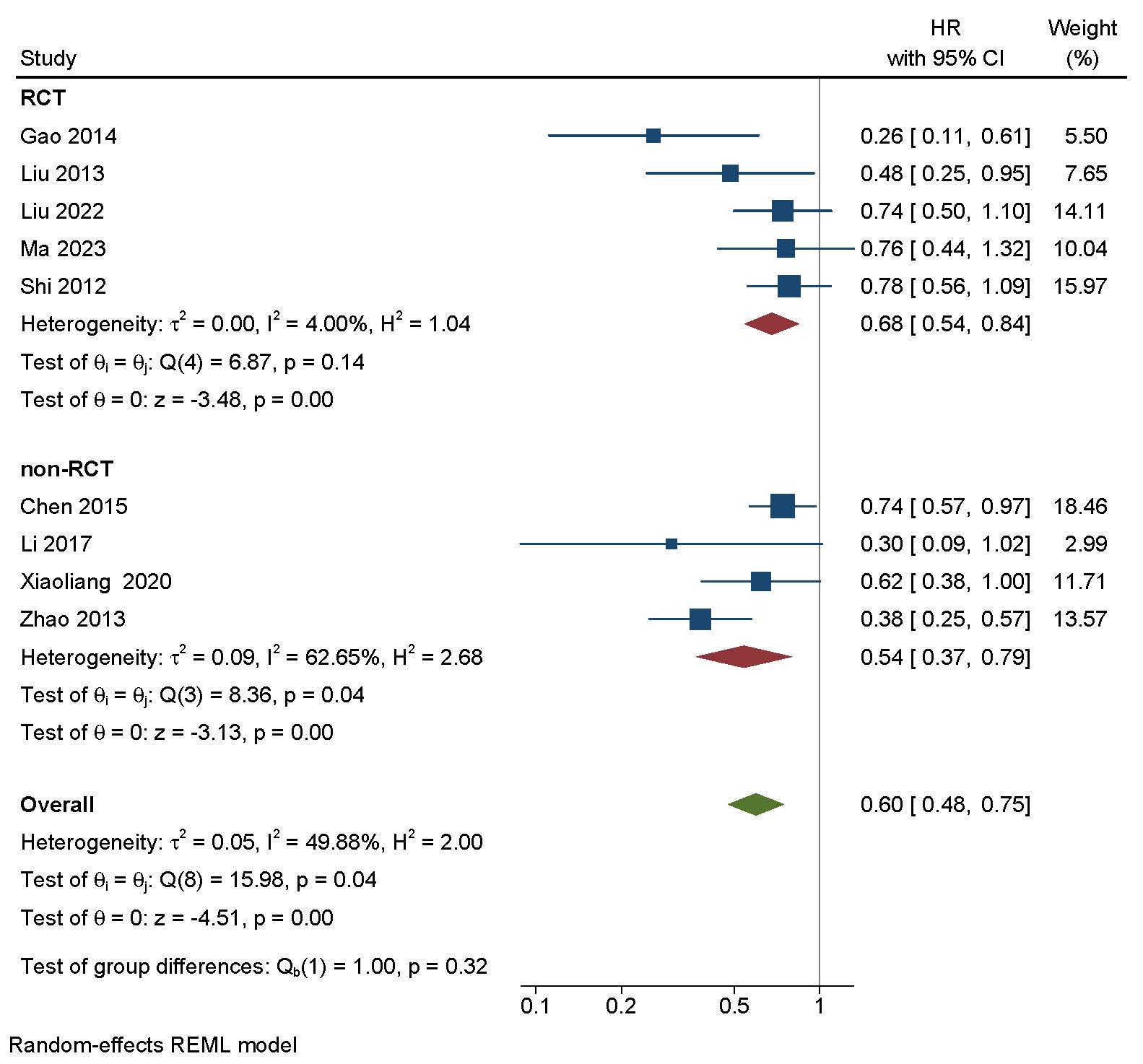


**Figure S6- Subgroup analysis of overall survival (OS) across randomized controlled trial (RCT) vs non-RCT studies. There is no significant difference between RCT and non-RCT studies regarding OS, however, there is still considerable heterogeneity among the non-RCT studies on CIK cells (I^2^ = 49.88%).**


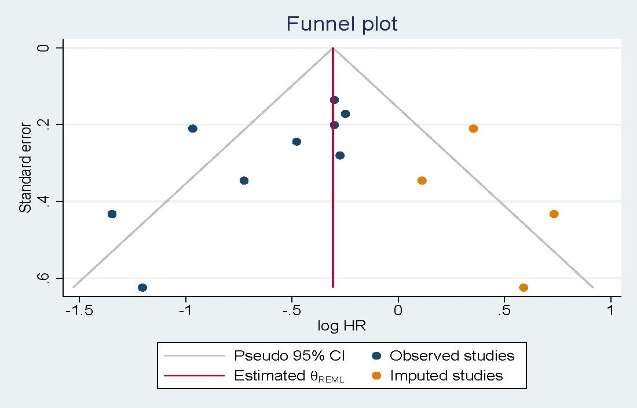

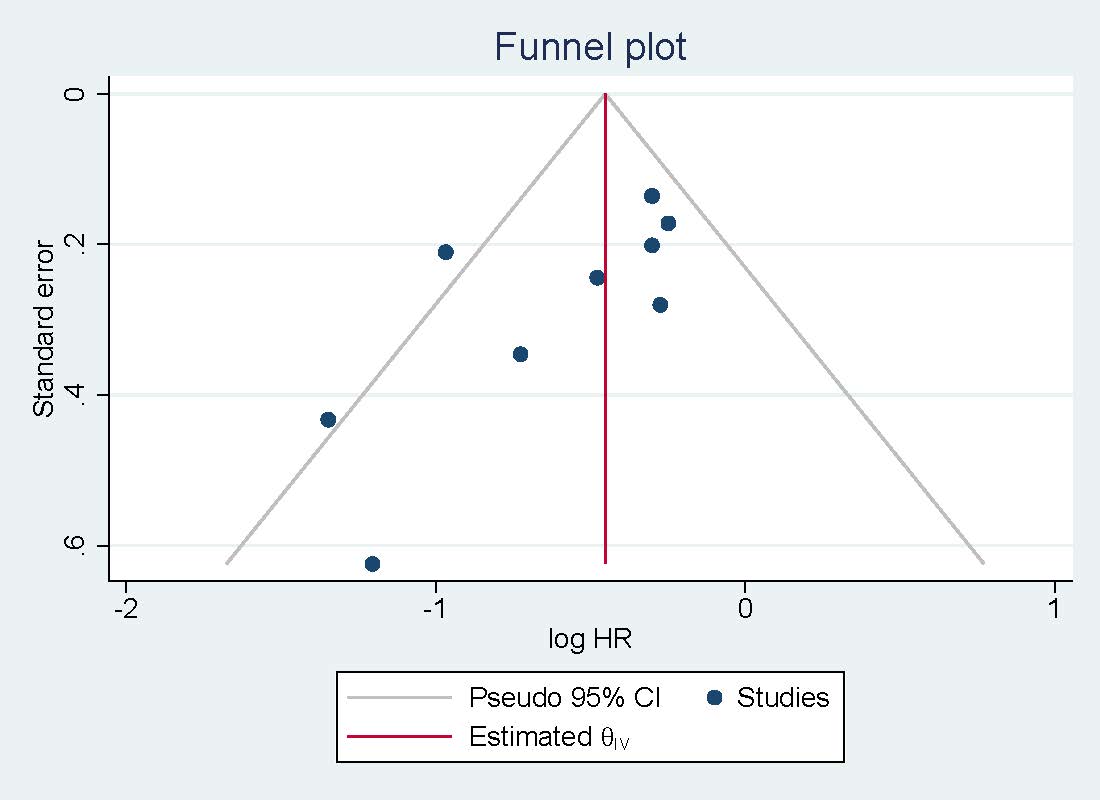


**A**

**B**

**Figure S7- (A) Funnel plot of assessment of publication bias considering overall survival (os) as the main outcome (B) Funnel plot with Trim and fill analysis for possible publication bias.**

**
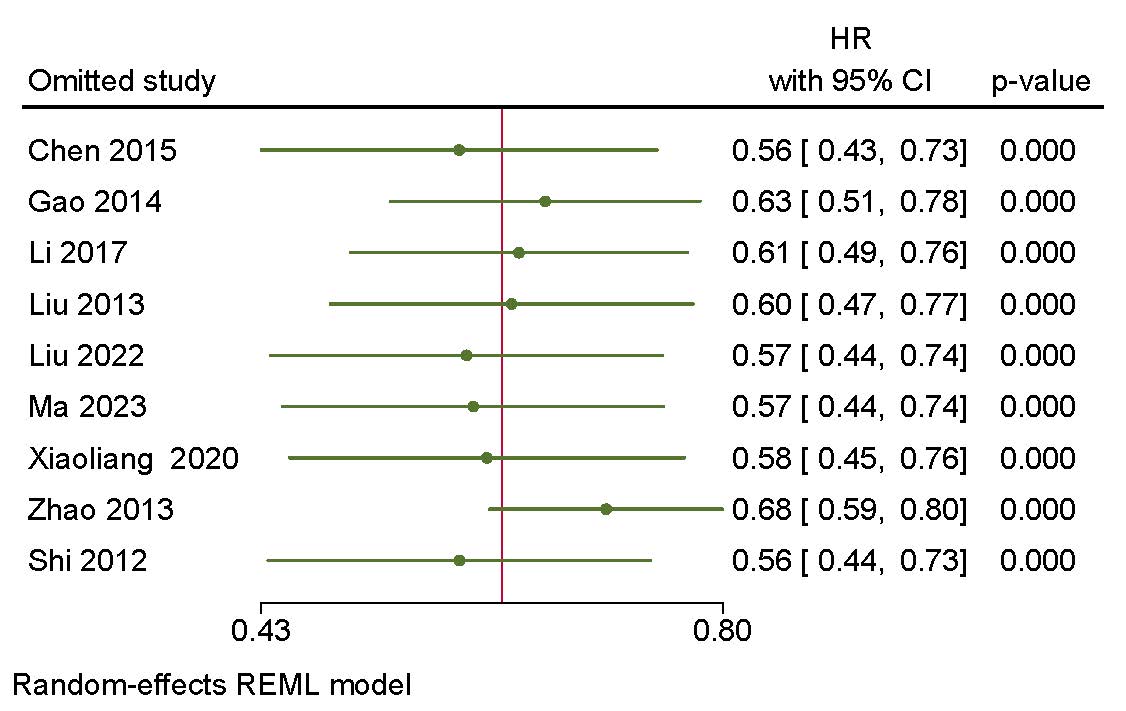
**

**Figure S8- Sensitivity analysis examining the influence of excluding each study on the pooled results of OS. In all the instances, the pooled effect size remains significant.**
